# A tool for simulating and communicating uncertainty when modelling species distributions under future climates

**DOI:** 10.1002/ece3.1319

**Published:** 2014-12-03

**Authors:** Susan F Gould, Nicholas J Beeton, Rebecca M B Harris, Michael F Hutchinson, Alex M Lechner, Luciana L Porfirio, Brendan G Mackey

**Affiliations:** 1 Griffith Climate Change Response Program, Griffith UniversitySouthport, Queensland, Australia; 2 School of Biological Sciences, University of TasmaniaHobart, Tasmania, Australia; 3 Antarctic Climate and Ecosystems CRCHobart, Tasmania, Australia; 4 Australian National UniversityCanberra, Australian Capital Territory, Australia; 5 University of TasmaniaHobart, Tasmania, Australia; 6 Griffith UniversitySouthport, Queensland, Australia

**Keywords:** Climate change, MaxEnt, measurement error, simulation, spatial ecology, spatial prediction, species distribution model

## Abstract

1. Tools for exploring and communicating the impact of uncertainty on spatial prediction are urgently needed, particularly when projecting species distributions to future conditions.
2. We provide a tool for simulating uncertainty, focusing on uncertainty due to data quality. We illustrate the use of the tool using a Tasmanian endemic species as a case study. Our simulations provide probabilistic, spatially explicit illustrations of the impact of uncertainty on model projections. We also illustrate differences in model projections using six different global climate models and two contrasting emissions scenarios.
3. Our case study results illustrate how different sources of uncertainty have different impacts on model output and how the geographic distribution of uncertainty can vary.
4. *Synthesis and applications*: We provide a conceptual framework for understanding sources of uncertainty based on a review of potential sources of uncertainty in species distribution modelling; a tool for simulating uncertainty in species distribution models; and protocols for dealing with uncertainty due to climate models and emissions scenarios. Our tool provides a step forward in understanding and communicating the impacts of uncertainty on species distribution models under future climates which will be particularly helpful for informing discussions between researchers, policy makers, and conservation practitioners.

## Introduction

Natural systems are inherently variable in both space and time. Consequently, models of natural systems, including species distribution models (SDMs), inevitably include some degree of uncertainty. Uncertainty is not problematic per se as long as its effects on model projections are not ignored. However, many correlative SDMs are spatially projected at fine resolution without explicitly addressing uncertainty, thereby implying a confidence in model outputs that may be misleading (Refsgaard et al. 2007; Sinclair et al. 2010; Beale and Lennon 2012; Wenger et al. 2013). Correlative SDMs are being widely used in conservation planning and to assess the adequacy of reserve systems under anticipated future climates. To reduce the risk of adverse conservation outcomes, it is important that any uncertainties in species distribution models are explicitly addressed. Explicitly addressing uncertainty is particularly important when projecting to future conditions as uncertainty increases the further removed a projection is from current conditions (Thuiller 2003). Broadly, three mutually compatible approaches for addressing uncertainty are available. The first approach is to reduce model uncertainty by increasing ecological knowledge and improving the modelling process. The second approach is to assess model uncertainty quantitatively or probabilistically. The third approach is to apply risk management measures that make decision making robust to model uncertainty. Irrespective of which approach is adopted, good scientific practice demands that model uncertainty is explicitly addressed and communicated.

Substantial progress has been made in reducing uncertainty in SDMs. Previous reviews have identified multiple sources of uncertainty (Guisan and Zimmerman 2000; Araújo et al. 2005; Guisan and Thuiller 2005; Barry and Elith 2006) and procedures for reducing their impacts (Vaughan and Ormerod 2005; Hernandez et al. 2006; Randin et al. 2006). Progress has also been made toward making conservation planning robust to uncertainties in SDMs (Moilanen et al. 2006; Carvalho et al. 2011; Bagchi et al. 2013). Recent advances have also been made in quantifying overall model uncertainty. However, the relative contribution of any single source of uncertainty to overall model uncertainty will vary with attributes of the model species, attributes of the landscape, size of the study area, and the location of the study area relative to climate projections. For example, Wenger et al. (2013) developed a probabilistic ensemble modelling approach for accounting for uncertainty in forecasts of species distributions under future climates. The current distribution of their model species, the bull trout, is already close to the limits of suitable climatic conditions. Consequently, differences between climate models accounted for most of the overall model uncertainty in their case study. In contrast, Dormann et al. (2008) compared the relative contributions of different sources of uncertainty in models of the great gray shrike under future climates. The study area, Saxony, was small, and there was little difference between the climate projections for the three emission scenarios modelled. In this case, model type and data quality accounted for most of the overall model uncertainty. Thus, there is no single best solution for minimizing uncertainty. This highlights the need for exploring and communicating uncertainty to be an explicit part of any modelling process.

**Figure 1:**
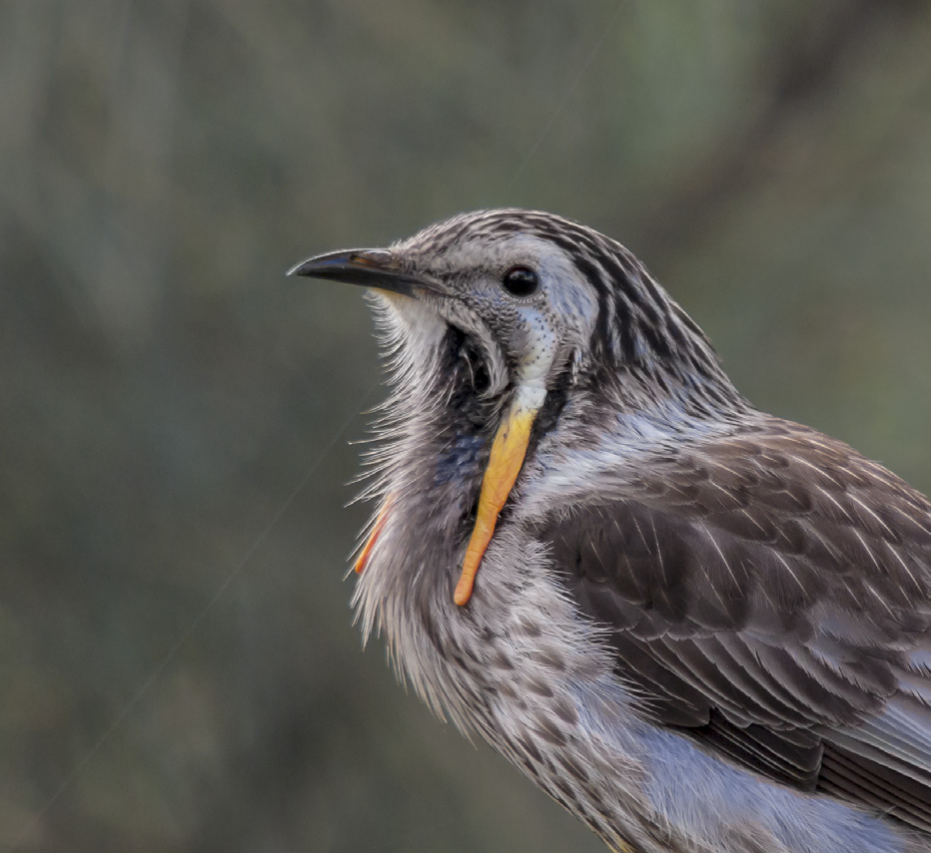
Our model species, *Anthochaera paradoxa* yellow wattlebird is a Tasmanian endemic (Photograph by Alan Fletcher)**.**

We provide a tool for simulating the effects of some known sources of uncertainty. The tool uses a Monte Carlo process to produce probabilistic, spatially explicit output. The simulation tool allows users to explore the impacts of different sources of uncertainty on spatial prediction. Furthermore, our tool provides a visual aid for communicating the impacts of uncertainty on spatial prediction. Communicating the impacts of uncertainty in a spatially explicit way could increase awareness of the potential impacts of uncertainty and reduce the risks that model outputs are misinterpreted (Elith et al. 2002; Wiens et al. 2009; Rocchini et al. 2011; Kujala et al. 2013). We illustrate the use of our tool by simulating known sources of uncertainty using a Tasmanian endemic, the yellow wattlebird, *Anthochaera paradoxa* (Daudin, 1800) as a case study (Fig. 1). We focus primarily on uncertainty relating to data quality and simulate the effects of locational uncertainty, spatial bias, uncertainty in climate data and model variance on spatial prediction. We also demonstrate how the choice of global climate model and emissions scenario can alter spatial prediction. Our modelling framework was developed in R, using the GTK+ toolbox to provide a graphical user interface for ease of use, and using MaxEnt (Phillips et al. 2006; Phillips and Dudik 2008) as the underlying species distribution model. The source is available in Appendix S1. The tool will be useful for simulating and communicating the impacts of some important sources of uncertainty on species distribution models.

## Uncertainty Defined

Uncertainty is a measure of unexplained variation that has three components in models of natural systems: (i) natural variability; (ii) measurement error; and (iii) incomplete knowledge about natural phenomena and complex processes. Natural variability is an inherent property of natural systems. Across a species’ distribution, populations can differ in morphology, movement behavior, and habitat preferences in response to local conditions. Species and ecosystems are also constantly adjusting to the contingencies of environmental drivers including climate. Ideally, natural variability would be classed as explained variation and accounted for separately from uncertainty (Lehmann and Rillig 2014). In practice, however, there are limitations to our ability to separately account for natural variability and it is usually included with other uncertainty. Measurement error includes various shortcomings that can arise when modelling species distributions including errors in input data. The magnitude of uncertainty due to measurement error can be probabilistically quantified. Uncertainty due to incomplete knowledge, including knowledge of future events, however, cannot be eliminated or quantified.

## Uncertainty Framework

**Figure 2:**
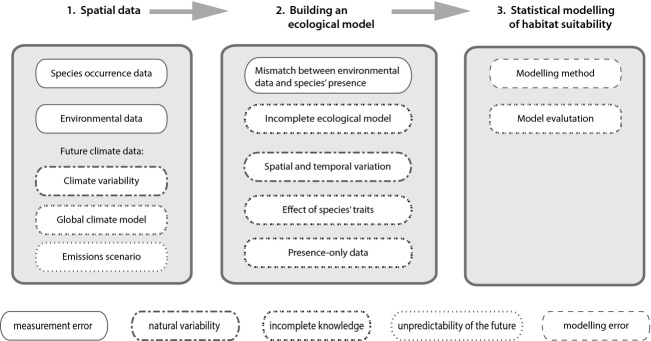
Potential sources of uncertainty in the species distribution modelling process. Different classes of uncertainty are indicated by the box borders.

Our conceptual framework for uncertainty in SDMs is outlined in Fig. 2. Some sources of uncertainty affect multiple steps in the modelling process. Our framework of uncertainty is structured according to where a given source of uncertainty first enters the modelling process beginning with the collation of spatial data. Our framework also indicates the class of uncertainty, that is, whether the uncertainty is due to measurement error, natural variability, incomplete knowledge, the unpredictability of the future, or modelling error. To supplement the conceptual framework, the potential sources of uncertainty are outlined in more detail in Table 1.

**Table 1:** Potential sources of uncertainty

**1. Input data**	
1.1 Species occurrence data: (i) positional errors; (ii) incorrect identification; (iii) truncated data; (iv) translocated species; (v) detectability; (vi) sampling bias	Elith et al. (2002); Kadmon et al. (2003)
1.2 Environmental data: (i) classification error; (ii) spatial interpolation error; (iii) incomplete data; (iv) instrument error; (v) rasterizing vector data	Lu and Weng (2007)
1.3 Future climate data: (i) climatic variability; (ii) GCM model differences; (iii) emissions scenarios	Beaumont et al. (2008); Daly et al. (2010)
**2. Building an ecological model**	
2.1 Spatial or temporal mismatch between input data and species’ ecology	Heikkinen et al. (2006); Dormann (2007); Roubicek et al. (2010)
2.2 Incomplete understanding of species’ ecology or inability to reflect ecological complexity: (i) specific habitat requirements; (ii) specific physiological requirements at different life stages; (iii) dispersal behavior; (iv) source–sink spatial structure	Pulliam (2000); Kearney (2006)
2.3 Effects of species traits on model accuracy: (i) range size; (ii) specialists cf. generalists; (iii) commonness	Stockwell and Peterson (2002); Kadmon et al. (2003); McPherson and Jetz (2007)
2.4 Spatial variation in species’ ecology due to the following: population-specific local optima and (ii) variation in limiting factors across species range	Urban et al. (2007); Rodder and Lotters (2010); Souther and McGraw (2011)
2.5 Temporal variation in species’ ecology due to the following: (i) development of nonanalogous environmental conditions; (ii) altered outcome of species interactions; (iii) adaptation and evolutionary change; (iv) phenotypic plasticity; (v) niche shifts	Davis et al. (1998); Pearson and Dawson (2003); Araújo and Luoto (2007); Suttle et al. (2007); Urban et al. (2007); Kissling et al. (2010); Montoya and Raffaelli (2010)
2.6 Use of presence-only data	Barry and Elith (2006); Phillips et al. (2009); Elith et al. (2011)
**3. Statistical modelling of habitat suitability**	
3.1 Modelling method including model parameterization	Segurado and Araujo (2004); Elith et al. (2006); Pearson et al. (2006); Elith and Leathwick (2009); Elith et al. (2010); Merow et al. (2013)
3.2 Model selection and evaluation	Araújo et al. (2005); Vaughan and Ormerod (2005); Allouche et al. (2006); Lobo et al. (2008); Rupprecht et al. (2011); Warren and Seifert 2011; Wenger and Olden (2012)

## Uncertainty Due to Spatial Data

Uncertainty relating to species observational data can be caused by sporadic errors such as taxonomic misidentification, inaccurate or imprecise locational data, or systematic error such as spatially biased sampling effort (Table 1). Although modern technology enables accurate recording of location, SDMs often use historical data which has unquantified location error or imprecise positional information. Almost all published models based on presence-only data are subject to spatial bias because data have not been collected systematically. Spatial bias in species location data is commonly an artifact of road distribution but can also be caused by environmental bias. In the absence of information to quantify spatial bias, most models incorrectly assume equal sampling effort across the modelled region.

### Current climate data

The impacts of uncertainty in climatic data on SDMs are rarely addressed but potentially significant (Willmott and Johnson 2005; Dobrowski 2011; McKenney et al. 2011). Uncertainty in climate data can result in inaccurate specification of the values of environmental variables used in SDMs (Bedia et al. 2013). The primary causes of uncertainty in current climate data are incomplete records and spatial interpolation. Uncertainty in spatial interpolation can be caused by the modifying effects of complex terrain, season, cloudiness, and geographical effects (Hutchinson 1991). Soria-Auza et al. (2010) demonstrated that uncertainty in climate data can cause large geographic discrepancies in model predictions for tropical species.

Uncertainty in SDMs can also be caused by the use of coarse-resolution climatic data. This uncertainty can be reduced by incorporating high-resolution terrain data in a spatial climate model (Hutchinson 1991; Daly et al. 2008; Fridley 2009; Hutchinson et al. 2009). This can provide finer-scale temperature estimates by incorporating temperature lapse rates associated with altitude. However, there are limits to the improvements that can be achieved, particularly in areas where meteorological data stations are sparsely distributed, and variables such as rainfall do not have simple relationships with orography. This is particularly important in coastal and mountainous regions (Hijmans et al. 2005; Daly 2006). The use of fine-scale digital elevation models without representation of local climate processes can lead to unwarranted confidence by creating an impression of greater accuracy than is justified by the underlying climate data (Mearns et al. 2003). Sharples et al. (2005) show that there are specific limits to the additional accuracy that finer-resolution topographic dependence can provide. Furthermore, the transferability of SDMs declines as the precision of climatic data increases, especially in regions where species range limits coincide with steep climatic gradients (Kriticos and Leriche 2010).

### Future climate data

Projected increases in global mean surface air temperature for 2100 range from 0.2–4.8°C, relative to 1990 (IPCC 2013). The main sources of uncertainty contributing to this wide range of values are as follows: (i) the natural variability of the climate system; (ii) uncertainty around future greenhouse gas emissions; and (iii) differences between global climate models (GCMs). Each GCM submitted to the Coupled Model Intercomparison Project (CMIP) archive is rigorously assessed, and only admitted if it provides a plausible representation of climate. Each individual GCM is deterministic in the sense that it calculates a specific repeatable result for a given set of input variables.

However, intramodel and intermodel differences arise because of model specification, resolution, and parameterization (Beaumont et al. 2008; Harris et al. 2014). In the short term, and at regional scales, the greatest sources of uncertainty in future climate data are due to differences between GCMs and natural climate variability. In the longer term, and at larger spatial scales, the major sources of uncertainty are associated with GCMs and emission scenarios (Harris et al. 2014).

Greenhouse gases and aerosols are a major influence on climate and another source of uncertainty. The degree of uncertainty about future greenhouse gas emissions increases with time. Changes projected under lower-emission scenarios are often qualitatively similar but smaller in magnitude than higher-emission scenarios, with scenarios only diverging in the latter part of the 21st century.

## Uncertainty Due to Model Specification

Ecological knowledge is critical to selecting meaningful and appropriately scaled variables for use in correlative SDMs (Austin 2002, 2007). Variable selection is potentially a source of uncertainty in the sense that model projections will vary according to the variables that are included in the model. Williams et al. (2012) have outlined a process for systematically selecting environmental variables for biodiversity modelling by examining relationships between a species’ ecological model, spatial environmental data and a statistical model. The lack of species absence data also affects the accuracy of SDMs as the use of presence-only data can lead to inaccurate identification of the attributes of unsuitable sites (Barry and Elith 2006; Phillips et al. 2009). Merow et al. (2013) illustrated the importance of ecological knowledge in specifying absence data. Ecological knowledge is also important for accurately matching species data with environmental data. Matching species’ presence data with environmental data both spatially and temporally is necessary for accurate specification of the ecological model. Accurate data matching is particularly important when modelling migratory species and when projecting to future conditions as shown by (Heikkinen et al. 2006). Baseline climatic data should also correspond to the time period in which the species data were collected (Harris et al. 2014).

## Statistical Modelling Method

Model selection and evaluation are problematic when projecting to future conditions as no independent evaluation data are available. A common approach is to compare the predictive performance of different modelling methods using the same input data. However, even with high individual evaluation scores, large discrepancies can exist between the spatial outputs of different methods (Loiselle et al. 2003; Thuiller 2003; Pearson et al. 2006; Rupprecht et al. 2011). Pearson et al. (2006) showed that the modelling method can have a large impact on the direction and magnitude of change. Failure to account for these sources of uncertainty can lead to spurious predictions of expansion or contraction of species’ distribution. Various methods are available for evaluating predictive performance, but they all suffer from a lack of systematically collected, independent evaluation data.

## Case Study: Mapping the Effects of Uncertainty on Spatial Prediction for *A. paradoxa*

To illustrate how uncertainty can affect spatial prediction, we modelled a Tasmanian endemic species, *Anthochaera paradoxa,* the yellow wattlebird, using MaxEnt (Elith et al. 2006; Phillips et al. 2006; Phillips and Dudik 2008). MaxEnt is widely used to model species distributions because it has been shown to perform well with presence-only data and because of its ease of use. All modelling methods, however, including MaxEnt have limitations. In the case of MaxEnt, the method for generating background samples from presence-only data is a source of variability (Merow et al. 2013). We first modelled the species without simulating uncertainty and then individually simulated the effects of different sources of uncertainty on spatial prediction using a Monte Carlo process. This process results in probabilistic spatial predictions of predicted presence. We interpret predicted presence as habitat suitability.

Species occurrence data for *A. paradoxa* were sourced from the Natural Values Atlas, Tasmania (Department of Primary Industries Parks Water and Environment 2014). The data were filtered to remove observations that were not contemporaneous with the current climate data, where current is defined as 1976 to 2005. This left 1517 records, of which 1339 were from the first Atlas of Australian Birds project. We are confident in the accuracy of species identification as the Atlas data were rigorously vetted. The locational data, however, have low precision. Species locations are reported as the coordinates of the center of a 10-minute, that is, approximately 18.5-km, grid cell (A. Silcocks, pers. comm.).

A logistic habitat suitability value was estimated using six bioclimatic variables that represent the mean, range, and seasonality of key components of climatic regimes: (i) annual mean temperature; (ii) minimum temperature of the coldest month; (iii) maximum temperature of the warmest month; (iv) annual precipitation; (v) precipitation in the warmest quarter; and (vi) precipitation in the coldest quarter. The values for these variables were derived from long-term mean monthly records of maximum and minimum temperature, and precipitation.

We generated estimates of current climate, that is, the period 1976 to 2005, centered on 1990, using ANUCLIM v6.1 (Xu & Hutchinson 2011). We then calculated climate change grids relative to 1990 for 2085 (i.e., the center of the period 2070 to 2099), for two emissions scenarios and six GCMs. High (A2)- and low (B1)-emission scenarios (IPCC 2000) were selected to bracket the range of values due to differences in emission scenario. A single iteration of each of six dynamically downscaled GCMs (ECHAM5/MPI-OM, GFDL-CM2.0, GFDL-CM2.1, UKMO-HadCM3, CSIRO Mk3.5 and MIROC3.2_medres) was used. These GCMs represent the means and variability of the current climate in southeastern Australia and cover the range of projected rainfall change in the CMIP3 archive (Corney et al. 2013). We used ANUCLIM to further interpolate the future dynamically downscaled climate data to a 1-km resolution and generate monthly mean data for the current and future periods.

Thus, for each individual simulated source of uncertainty, there were 13 outputs, one for current climate and 12 for 2085. For the purposes of brevity, we only present results for the A2 emissions scenario and the two GCMs that best illustrated the need to consider uncertainty due to climate model. In this case, the GCMs that were most different were CSIRO Mk3.5 and GFDL-CM20. Additional results are provided in Supporting Information.

## Locational Uncertainty

We simulated uncertainty in the locational data, a type of measurement error (Fig. 2), by adding an average of 10 km of normally distributed noise to each point of species data, and then running the model on the modified data (see Supporting Information). This ensured that most perturbed points were within the specified locational accuracy of the bird data while covering the range of possibilities. We repeated this process 100 times to generate a suite of expected distributions. Thus, we estimated each cell's probability of being classed as a “presence” given the locational uncertainty.

**Figure 3:**
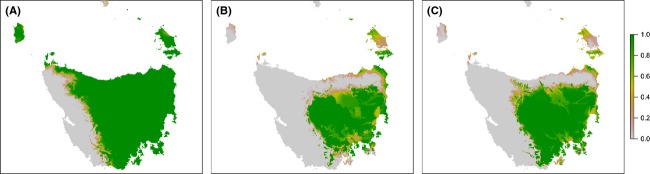
Comparison *of Anthochaera paradoxa* models with simulated uncertainty in locational data for (A) current climate; and projected climate in 2085 under the A2 scenario using climate models (B) CSIRO MK3.5; and (C) GFDL-CM20. Each plot shows the proportion of model runs predicting species presence.

Under current climate, the impacts of locational uncertainty were concentrated in the northwest and along the western limit of the modelled distribution (Fig. 3A). Model sensitivity to locational uncertainty is greatest where relatively small changes in location are equivalent to changes in the values of variables in the model, causing a transition over the model threshold which separates “presence” from “absence”. That is, model sensitivity to locational uncertainty is greatest in areas where the model also predicts that habitat suitability is marginal. Model output for the future time step indicated even further contraction of the area where climatic conditions are potentially suitable (Fig. 3B and C).

The impact of locational uncertainty is likely to be similar in magnitude to the impact of uncertainty due to misclassification of environmental data. However, error due to incorrect species identification or translocation of individuals could result in anomalous data that greatly exceed the direction and magnitude of error compared to the nominal 10-km average locational error illustrated here.

## Spatial Bias

**Figure 4:**
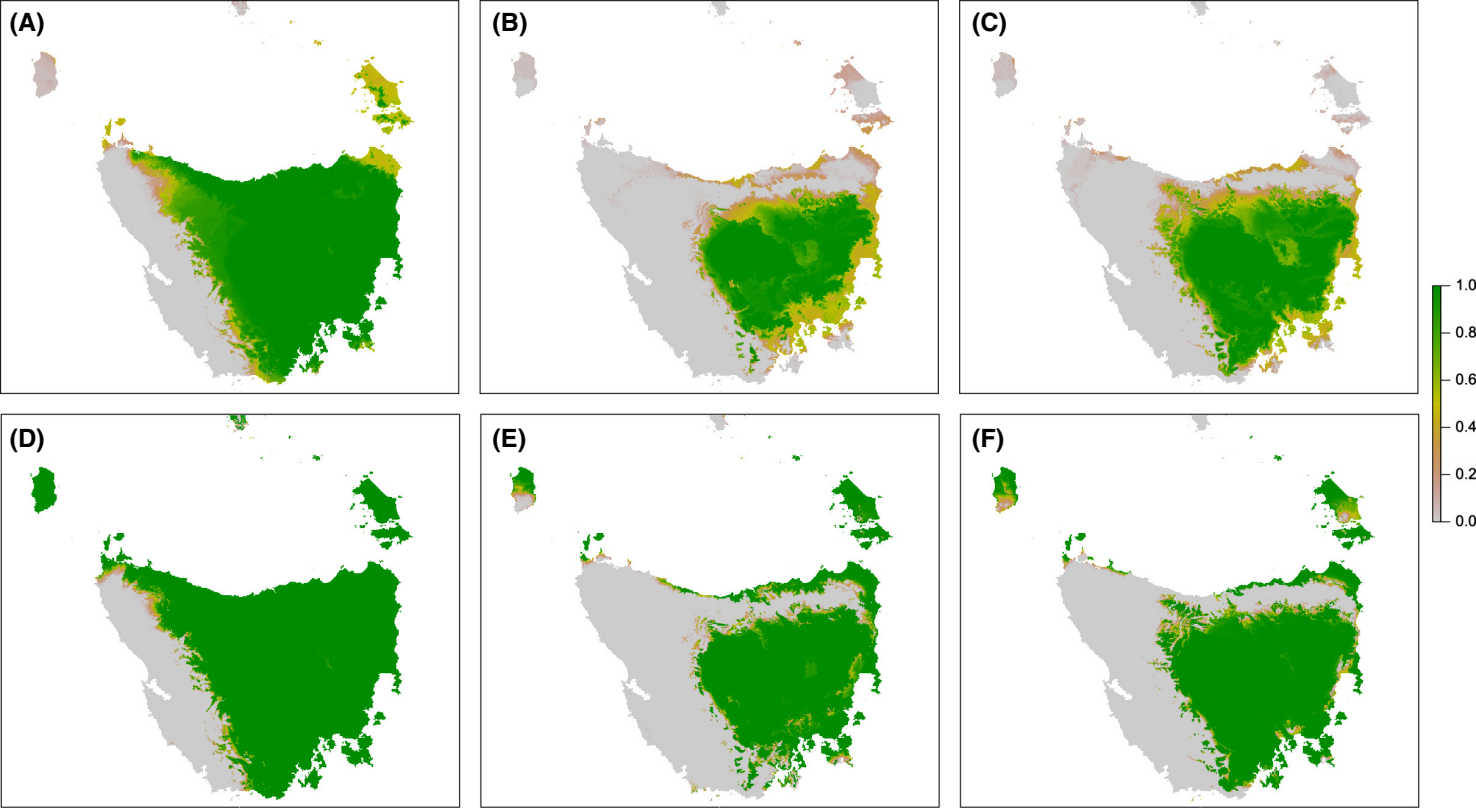
Comparison of *Anthochaera paradoxa* models showing the impact of spatially biased data loss and random data loss on spatial prediction. The model with spatially biased data loss is shown for (A) current climate; and projected climate in 2085 under the A2 scenario using climate models (B) CSIRO MK3.5; and (C) GFDL-CM20, followed by the model with random data loss for (D) current climate; and projected climate using (E) CSIRO MK3.5; and (F) GFDL-CM20 showing the proportion of model runs predicting species presence.

We simulated the impact of spatial bias, a type of measurement error (Fig. 2), using nonrandom cross-validation by repeatedly removing the 10% of spatially autocorrelated data most distant from a randomly assigned point. We compared the impact of spatial bias with the impact of random loss of 10% of the dataset. Spatial bias had a much larger impact on model projection than random data loss although the absolute number of data points was the same. In the presence of a small amount of spatial bias, the number of predicted presences declined substantially (Fig. 4) and a large part of the range was affected. With spatial bias, areas that were otherwise core habitat became marginal habitat for current conditions and unsuitable habitat in the future. This occurs because the geographic distribution of the impact of spatial bias is determined by the way in which the spatial bias skews the values of the covariates used in the model (Elith et al. 2011).

This simple simulation illustrates the amount of uncertainty we could expect with a small amount of spatial bias in sampling effort. Thus, a small unbiased dataset may be preferable to a larger but spatially biased dataset. This simulation may be indicative of the magnitude of uncertainty we could expect due to various sources of spatial bias caused by spatial and temporal variation (Fig. 2). For example, a change in limiting factors across a species’ range, variation in species detectability in different habitats, truncated species data, and population-specific local optima are all potential causes of spatial bias in the species data.

Nonrandom cross-validation has also been used to evaluate model transferability (Wenger and Olden 2012). The large impact of quite conservative amounts of simulated spatial bias on the *A. paradoxa* model indicates that the model is unlikely to transfer well to a different dataset, a different location, or a different time period; that is, the model lacks generality (Vaughan and Ormerod 2005; Randin et al. 2006; Wenger and Olden 2012). Furthermore, it may indicate that validation of this model using standard procedures, which use nonindependent data, may overestimate the predictive ability of the model (Araújo et al. 2005). Thus, as the time frames of future projections increase, the more circumspect we should be in accepting the model outputs.

## Uncertainty in Climatic Data

We estimated uncertainty in the current climate grids, a type of measurement error due to interpolation (Fig. 2), by producing an error surface for each variable. The error surfaces consisted of rasters of spatially distributed standard errors for 36 variables (12 months × 3 variables). The rasters combined measurement and interpolation error in a way that approximately accounted for spatial correlation in the gridded climate values. The rasters were generated using code adapted from the ANUSPLIN thin-plate smoothing spline software package (Hutchinson and Xu 2013) to take account of all spatially random errors and their spatial correlation. Perturbation grids were independently simulated at 0.5°C resolution for monthly minimum temperature and monthly maximum temperature, and at 0.25°C for monthly precipitation. These were identified as the minimum resolutions where spatial correlation between errors became negligible. These values provide a direct measure of the differing spatial scales of interaction with topography of monthly mean temperature and monthly mean precipitation. We then sampled from a normal error distribution for each cell with standard deviation specified by the grid and used bicubic interpolation to apply this error to the original climate surfaces. Spatial correlations between the climate variables were not modelled. While there are modest correlations between daily time series values of these variables, these correlations are largely removed when integrated to 30-year monthly means and considered in the context of spatial interpolation errors. This is confirmed in particular by the differing spatial scales of interaction with topography exhibited by monthly mean temperature and monthly mean precipitation.

**Figure 5:**
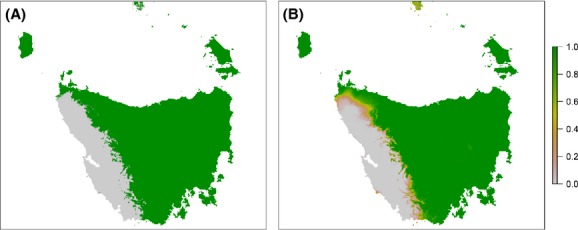
Comparison of *Anthochaera paradoxa* models showing (A) the original model with no simulated uncertainty and (B) a perturbed model showing the effects of simulated current climatic uncertainty. In (A), green denotes presence and gray absence, whereas (B) shows the proportion of the 100 model runs predicting species presence in each cell.

The impact of uncertainty in current climate data on spatial prediction was similar in magnitude to locational uncertainty (Fig. 5). The simulated climatic uncertainty resulted in a contraction of core habitat and an expansion of marginal habitat. The magnitude of impact of uncertainty in future climatic projections will necessarily be much higher than that shown for current conditions as it depends not only on the accuracy of the climatic data but also on additional sources of uncertainty that are introduced by projecting to future climates.

## Model Variance

**Figure 6:**
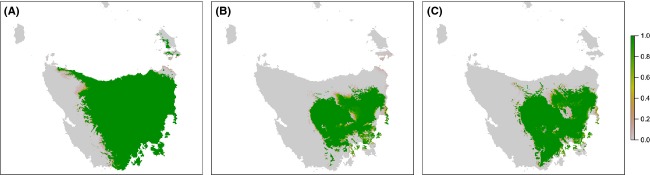
Comparison between *Anthochaera paradoxa* models showing uncertainty due to model variance for (A) current climate; and for projected climate in 2085 under the A2 scenario using climate models (B) CSIRO MK3.5; and (C) GFDL-CM20 showing the proportion of model runs predicting species presence.

Statistically speaking, model variance is that part of a model's total error that is explained by the effect of variation in the training data (De'ath 2007). We estimated model sensitivity to the dataset, a type of modelling error (Fig. 2) using cross-validation. This method has been shown to be robust for small datasets which are common in many SDMs. The dataset was split into 100 equal segments; then, each segment was removed one at a time to test the model generated by the remaining 99 segments. This process created 100 separate distributions, each of which was relatively unbiased, having been tested using separate data from the training data. Differences between these models are due to model variance. The impact of model variance on model output was a contraction of the area predicted as suitable habitat. The magnitude of impact of model variance was greater than locational uncertainty or current climatic uncertainty but less than spatial bias (Fig. 6).

## All Simulated Sources Combined

We combined all sources of uncertainty by simultaneously applying all described methods of simulating uncertainty to the observation and climate data. The combined uncertainty included model variance but not differences between climate models or emissions scenarios. To combine model variance with the other simulated sources of uncertainty, a different approach was required as each model replicate is likely to contain different data both for the species and climatic data due to other sources of uncertainty. Consequently, when combining all simulated uncertainty, we instead used a random training–testing split for each model run to test model variance. This differs from cross-validation in that the testing sets are not mutually exclusive from run to run and is closer to a bootstrapping approach. However, we expect this method should produce similar results.

**Figure 7:**
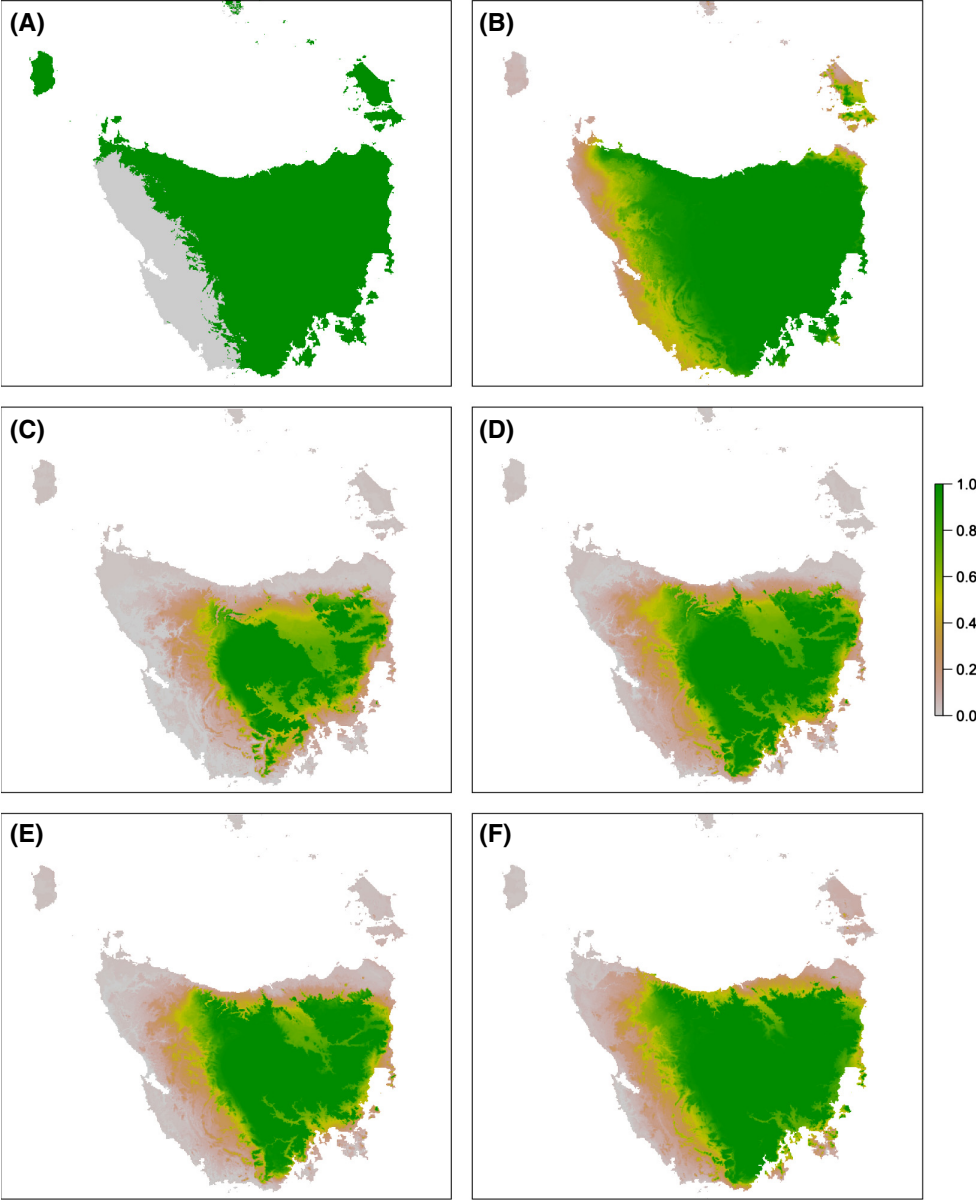
Comparison of *Anthochaera paradoxa* models showing (A) the original model for current conditions with no simulated uncertainty, with models including all simulated sources of uncertainty combined for (B) current conditions, and projected climate in 2085 using climate models (C) CSIRO MK3.5 under the A2 scenario; (D) GFDL-CM20 under the A2 scenario; (E) CSIRO MK3.5 under the B1 scenario; and (F) GFDL-CM20 under the B1 scenario. In (A), green denotes presence and gray absence, whereas all other figures show the proportion of model runs predicting species presence.

When all simulated sources of uncertainty were combined, the uncertainty in the spatial distribution was larger than any individual source of uncertainty (Fig. 7). The model that combined all simulated sources of uncertainty regularly predicted suitable habitat in areas that were predicted as unsuitable habitat in the model with no simulated uncertainty. Conversely, the model that combined all simulated sources of uncertainty predicted poor habitat suitability in areas that were predicted to be core habitat in the model with no simulated uncertainty. Taken together, these observations suggest that the effects of uncertainty not only blur the margins of a predicted distribution, but can also skew the result. There were substantial differences in model predictions between emission scenarios and global climate models (Fig. 7). Differences between scenarios appear to be mainly in the magnitude of change. However, differences between the two GCMs, which represented the extremes of the six GCMs considered, varied in the direction of change.

The examples illustrated here represent modest and realistic levels of uncertainty. All of these sources of uncertainty and more are likely to be present but unaccounted for when modelling species distributions. Our simulations should provide reasonable estimates of the actual uncertainty in the species distribution model if for any given source of uncertainty the actual level of uncertainty is low and well-defined. As the level of actual uncertainty increases, however, the more the distributions of simulated uncertainty will diverge from the actual but unknown distribution of uncertainty. In most cases, it is safest to interpret the results as generalized illustrations of the effects of uncertainty rather than as reliable distribution maps.

## Discussion

Our case study results illustrate three important points. Firstly, the spatial distribution of uncertainty is not homogeneous and can vary substantially across a species’ predicted habitat. Secondly, the way that the uncertainty is spatially distributed depends on how the uncertainty impacts the model specification. While the general effect of uncertainty is to move predictions closer to the model threshold and thus blur the edges, spatial bias can skew the values for the covariates in the model so that when it is projected spatially, it results in different geographic output. Thirdly, the combined effects of different sources of uncertainty are greater than the effect of any individual source of uncertainty. Thus, simulating just one source of uncertainty, even if it has a large effect, may not be sufficient to represent other sources of uncertainty.

In the case of *A. paradoxa*, simulated spatial bias in the species data had the greatest impact on spatial prediction. Spatial bias is one source of uncertainty that is usually present but rarely accounted for in SDMs. However, comparison of studies that have quantified overall uncertainty shows that the relative magnitude of an individual source of uncertainty is likely to vary from one case to another (Dormann et al. 2008; Wenger et al. 2013). Factors impacting the relative magnitude of individual sources of uncertainty are data quality, species and landscape attributes, the size and location of the study region with respect to future climate projections, and the future climate projections for the study area.

Our simulations illustrate that uncertainty can substantially affect spatial prediction. This emphasizes the need to address uncertainty as an explicit part of the experimental protocol for modelling species distributions. Our simulation tool provides a potentially valuable tool for communicating the impacts of uncertainty on spatial prediction. As the tool provides spatially explicit output, it could be a powerful aid in the ongoing dialogue that should be taking place between researchers, policy makers, and practitioners. Furthermore, our tool provides a method for evaluating model transferability. Evaluating transferability is particularly important when projecting species distributions under future climates as independent test data are not available.

Different approaches are needed when uncertainty is due to incomplete knowledge including our inability to predict the future. To reduce known sources of uncertainty when projecting to the future, GCM selection should be based on which models best represent key environmental variables for the model species and the study region. For example, many species’ distributions are strongly driven by temperature and rainfall, but some GCMs project “wetter” or “hotter” than the mean of all GCMs, and these projections vary spatially. In southeastern Australia, for example, MIROC3.2_medres projects a climate that is wetter and cooler than the mean of all models in the CMIP archive, while the ECHAM5 model is warmer and drier (Harris et al. 2014). If increased rainfall is known to have a negative impact on a species of interest, GCMs that project increased precipitation in the study region could be used to assess the worst-case scenario when developing SDMs under future climatic conditions. Irrespective of what approach is used, the GCMs selected must be explicitly stated, to assist interpretation of the results. The best available method for assessing uncertainty due to GCM is to use multiple CMIP GCMs within an ensemble approach. Multimodel means (MMM) are sometimes used but do not represent the variability of the input models, seasonal variation, or daily extremes. Using MMMs can therefore conceal uncertainty (Beaumont et al. 2007). Furthermore, averaging individual variables can produce results which are physically implausible (Knutti et al. 2010) and unlike any individual model. If a MMM approach is used, we recommend the additional use of individual GCM inputs to assess the range of variation due to climate model.

The best way to represent uncertainty due to future greenhouse gas emissions is to model a range of plausible emissions scenarios, for example, SRES emissions scenarios A2 and B1, or RCP 2.6 and 8.5 (IPCC 2007). Including the upper limits of proposed emission scenarios may be important for conservation planning where assessments of potential impacts are guided by the precautionary principle. Modelling emission scenarios that bracket plausible futures will indicate the amount of uncertainty that is due to future human actions.

There remain a number of potential sources of uncertainty that cannot be quantified or bracketed to show differences between plausible model outputs, for example, uncertainty due to incomplete knowledge about specific habitat requirements, or how the outcomes of biological interactions will change as the climate changes. To address unquantifiable uncertainty, we recommend that all potential sources of uncertainty should be systematically reported along with model outputs. Furthermore, to maximize the transparency of the modelling process and enable independent assessment of model outputs, all parameterizations should be reported including the following: which GCMs and emission scenarios are used, how GCMs have been parameterized, downscaling methods, time frames for species data, and baseline climate data. Finally, to minimize linguistic uncertainty, it should be clearly stated what is being spatially projected. Models based on correlative analysis of climatic variables predict a species’ potential climate domain. They do not account for other environmental and ecological factors that influence species’ distributions. At best, they represent potential habitat suitability. Nevertheless, as long as their limitations are understood, correlative species distribution models currently provide the best available tool to support conservation planning and management.

## Supporting Information

**Appendix S1.** Detailed description and discussion of the uncertainty simulation process (NB: Appendix includes “Figure 1 Matrix of uncertainty in six global climate models”).

**Data S1.** SDM Uncertainty Simulation Tutorial.

**Data S2.** Code for SDM Uncertainty Simulation.
