# Supplementary material for: A tool for simulating and communicating uncertainty when modelling species distributions under future climates
Source: Ecol Evol. 2014 Dec 3;4(24):4798–811. doi: 10.1002/ece3.1319 (PMC4278828; doi:10.1002/ece3.1319)
Supplement: Supplementary file 1 — Appendix S1. Detailed description and discussion of the uncertainty simulation process (NB: Appendix includes “Figure 1 Matrix of uncertainty in six global climate models”). [file ece30004-4798-sd1.docx]

**Appendix S1**

*Detailed description and discussion of the uncertainty simulation process*

To illustrate the effects of uncertainty on species distribution modelling, a modelling framework was developed in R, using the GTK+ toolbox to provide a graphic user interface for ease of use, and using MaxEnt ([Phillips, Anderson & Schapire, 2006](#_ENREF_1); [Phillips & Dudik, 2008](#_ENREF_2)) as the underlying species distribution model.

The modelling framework is based upon a Monte Carlo process. One or multiple sources of uncertainty associated with the input data are simulated. The data with simulated uncertainty is then repeatedly applied until a distribution of output maps is achieved. If sufficient runs of the Monte Carlo process have been performed, this distribution will represent the range of possibilities for the distribution map given that the assumptions made by the species distribution model are correct, and that the assumptions made regarding the level and types of uncertainty are correct.

*Assumptions and limitations*

In each case, for the purposes of illustration we assume that the original unmodified species observation data and spatial data are free from uncertainty, and that the uncertainty that has been simulated by modifying one or more inputs to the model is representative of actual observed uncertainty. With some cases of simulated uncertainty, we can also look at this in a more realistic sense – that is, treating the original observation and spatial data as imperfect samples of some theoretical “perfect” set of truly representative, uncertainty-free data. In these specific cases, an estimated probability distribution of the original data can be calculated by applying the known level of uncertainty to the original “imperfect” sampled point.

For example, if we know that the exact position of an observation is (*x*, *y*) but the process of observation, measurement and recording is imperfect and adds a known normally distributed error in both the x- and y-coordinates, then the probability distribution of the sampled point at given values (*x’*, *y’*) is

P( sample at (*x’*, *y’*) | original at (*x*, *y*) ) = N(*x’*; *x*, *s*^2^) N(*y’*; *y*, *s*^2^) **(eqn 1)**

where N(*x’*; *x*, *s*^2^) is the probability density of *x’* in a normal distribution with mean *x* and variance *s*^2^.

In reality, the exact position of the original observation will not be known because we only have access to the imperfect sample data. In this case, we can manipulate the above equation:

P( sample at (*x’*, *y’*) | original at (*x*, *y*) ) = N(*x’* - *x*; 0, *s*^2^) N(*y’-* *y;* 0, *s*^2^) **(eqn 2)**

And then using Bayes’ theorem:

P( original at (*x*, *y*) | sample at (*x’*, *y’*) )

= P( sample at (*x’*, *y’*) | original at (*x*, *y*) ) P( original at (*x*, *y*) ) / P( sample at (*x’*, *y’*) )

= N(*x’* - *x*; 0, *s*^2^) N(*y’-* *y;* 0, *s*^2^) P( original at (*x*, *y*) ) / P( sample at (*x’*, *y’*) ) **(eqn 3)**

where the normalising factor in the denominator can also be expressed as:

P( sample at (*x’*, *y’*) ) = ∫ P( sample at (*x’*, *y’*) | original at (*u*, *v*) ) P(original at (*u*, *v*)) d*u* d*v* **(eqn 4)**

If we assume a uniform prior probability for the location of the original data point P( original at (*x*, *y*) ), then equation 3 simplifies to:

P( original at (*x*, *y*) | sample at (*x’*, *y’*) )

= N(*x’* - *x*; 0, *s*^2^) N(*y’-* *y;* 0, *s*^2^)

= N(*x’* - *x*; 0, *s*^2^) N(*y’-* *y;* 0, *s*^2^) as the normal distribution is even

= N(*x’*; *x*, *s*^2^) N(*y’*; *y*, *s*^2^) **(eqn 5)**

which is the same probability distribution as in equation 1, but with the places of original and sample points swapped. As a result, the probability distribution of the original dataset – and by extension, the generated uncertainty map – will be the same as the probability distribution for data with our simulated uncertainty and subsequent uncertainty map.

However, this convenient duality property does not hold for most modelled sources of uncertainty. For example, say that we simulate a certain amount of simulated spatial bias on a known unbiased dataset. We cannot expect the uncertainty resulting from this process to be the same as when we apply it to a spatially biased subsample of the unbiased data. The model we use here to simulate spatial bias entails selecting a point from the original observation data at random (call this point (*x**,*y**)), and discarding the furthest points from this point up to a specified proportion of the sample, thus defining some threshold distance *d*. This can be defined by:

P( sample at(*x*,*y*) | original at (*x*,*y*) )

= 1 if √( (x - x*)^2^ + (y - y*)^2^ ) < *d,*

or 0 otherwise **(eqn 6)**

The converse of this can be defined as

P( original at (*x*,*y*) | sample at (*x*,*y*) ) = 1 **(eqn 7)**

which is self-evident. That is, if the data point exists in the sample, it must also exist in the original dataset. This doesn’t tell the whole story; we know that there are also points in the original dataset that are not in the sample at all, i.e.

P( original at (*x*,*y*) | no sample at (*x*,*y*) ) > 0 **(eqn 8)**

We cannot explicitly define this non-zero function, and thus calculate the spatially explicit uncertainty in the derived probability distribution, without complete knowledge of the original dataset or introducing bias - so in this case we are unable to calculate a reasonable probability distribution for the original dataset given the sample dataset.

*Methods*

**Uncertainty in locational data**

Uncertainty in point-based observation data is modelled assuming normally distributed uncertainty with mean zero and standard deviations independently in both axes in the given projected space (in our example, MGA Zone 55). The level of modelled uncertainty is here given as an average distance. The probability distribution of distance is thus given by

√( N(0, *s*^2^ )^2^ + N(0, *s*^2^ )^2^ ) = *s*χ_2_

where χ_2_ is the chi distribution with two degrees of freedom. Given that the mean *μ* of this distribution is given by *μ* = √2 Γ(1.5)/Γ(1), then the required standard deviation *s* for simulating the uncertainty in point-based observation data can be calculated given the mean distance of uncertainty *d* by

*s* = Γ(1)/(√2 Γ(1.5) ) *d*

**Spatially biased data loss and random data loss**

Described in detail in the main article.

**Climatic uncertainty**

Described in detail in the main article

**Model variance**

Described in detail in the main article

*Conclusion*

If, for a given source of uncertainty, the level of uncertainty is low and well-defined, then our simulated uncertainty should fairly closely resemble the actual unknown uncertainty. In this case, our modelled uncertainty is likely to provide a reasonable estimate of the actual but unknown uncertainty in the species distribution model. For some sources of uncertainty, this correspondence is in fact perfect. As the level of uncertainty increases, however, the more different the distributions of these two cases, i.e., simulated compared with actual but unknown uncertainty will be. We attempt to quantify the degree of divergence between the two cases by calculating the differences between maps of increasing levels of uncertainty. In most cases, it is safest to take the results as generalized illustrations of the effects of uncertainty rather than as reliable distribution maps for the specific case.

Phillips, S. J., Anderson, R. P. & Schapire, R. E. (2006) Maximum entropy modeling of species geographic distributions. *Ecological Modelling,* **190,** 231-259.

Phillips, S. J. & Dudik, M. (2008) Modeling of species distributions with Maxent: new extensions and a comprehensive evaluation. *Ecography,* **31,** 161-175.


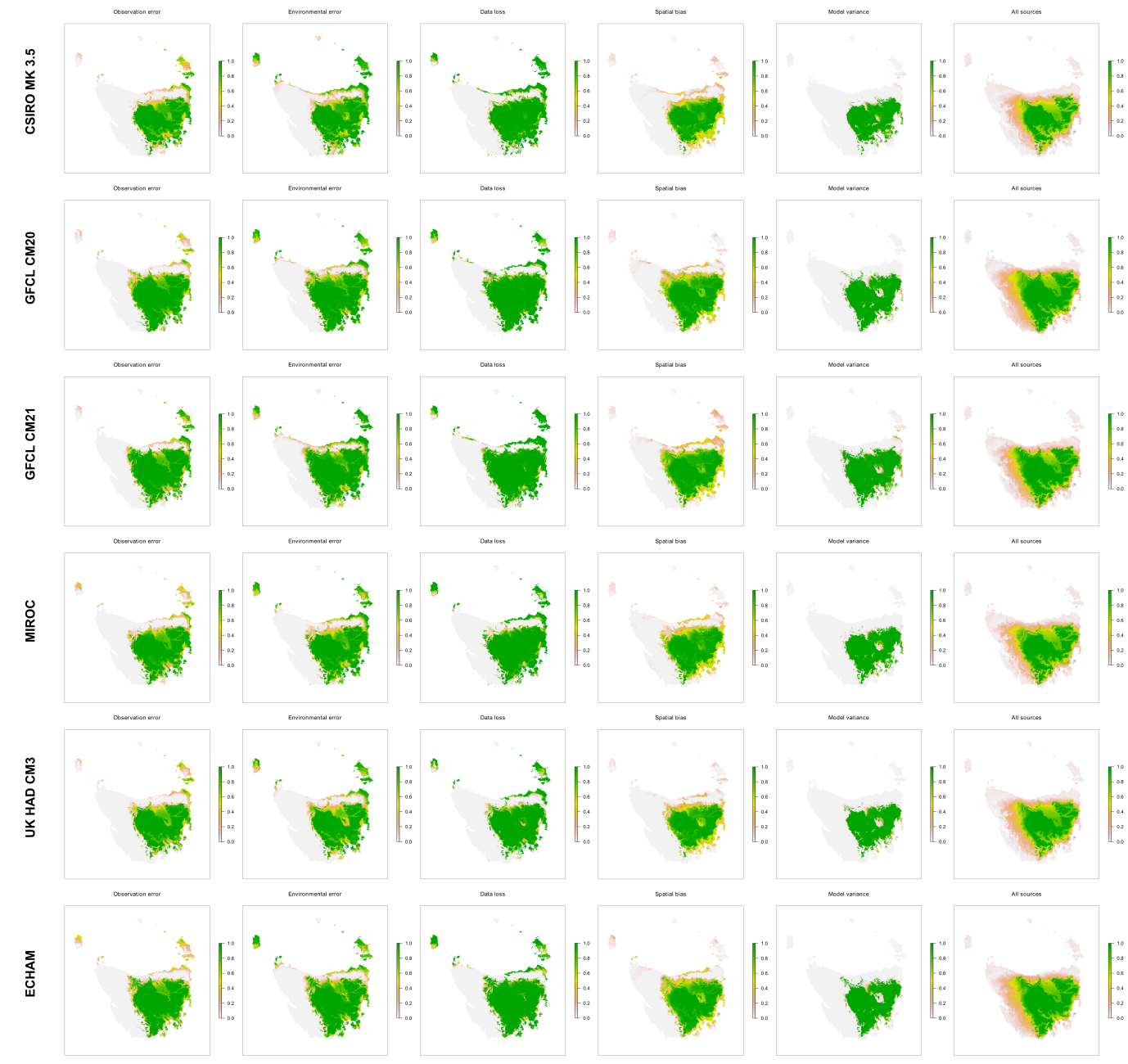


Figure 1 Matrix of uncertainty in six global climate models
