# Supplementary material for: A tool for simulating and communicating uncertainty when modelling species distributions under future climates
Source: Ecol Evol. 2014 Dec 3;4(24):4798–811. doi: 10.1002/ece3.1319 (PMC4278828; doi:10.1002/ece3.1319)
Supplement: Supplementary file 2 — Data S1. SDM Uncertainty Simulation Tutorial. [file ece30004-4798-sd2.docx]

# SDM Uncertainty Simulation Tutorial

## Installing software

Before running simulations using the provided code, you will need to download and install some software to allow you to run it. Maxent is available at <http://www.cs.princeton.edu/~schapire/maxent/> and the current version of R can obtained at <http://cran.r-project.org>. The code has been tested on R version 3.0.3 64-bit for Windows, and is currently not guaranteed to work on other versions or platforms, though it should with minimal fuss. To get Maxent to work, you may have to install Java, which is freely available at <https://www.java.com/en/download/>.

## Spatial Data Inputs

To run the uncertainty simulation, you will need the same data that you would use when running Maxent – specifically, observation data for one or more species and environmental data in raster form covering the region of interest.

### Point data

- same projection as environment data and covered by environment data
- preferably in latitude-longitude form
- .csv file with headers and three columns, representing Species, Long and Lat information respectively. Extra columns are fine – tell Maxent to ignore them when it prompts you – as long as the first three columns are correctly formatted as described
- As an example, we include *YellowWattleBird.csv* as used in the article

### Raster data

- ESRI ASCII file .asc format
- all using same projection and covering same area
- together in a single directory containing nothing else
- As an example, we include folders *bio.current* (BIOCLIM data) and *sdm.current* (monthly rainfall/temp data) which contain current climate information for Tasmania, and *bio.a2.mk3_5* which contains projected climate for Tasmania to 2070-2099 using the CSIRO MK3.5 Global Climate Model

### Climate error data (optional)

- ESRI ASCII file .asc format
- Is described in more detail later in this appendix and in the main article

## Running the program

### Setup

Before running the program, first open it in a text or source code editor of your choice and edit the java.dir and maxent variables (the writing inside quotation marks on these lines) to reflect the folder in which you have Java and Maxent installed respectively. These are defined in the first few lines of the code so should be easily found.

For running models in other regions than eastern Australia, you may also want to change the projection used – this is in line 644 on the code where the variable user.projection is defined. If you know what projection is best suited to your region of interest, replace the information in quotation marks on this line with the proj4 data for your projection (you can find this at <http://spatialreference.org> if you don’t know it).

***Starting the program***

Load R, then go to “File 🡪 Source R code…” and choose the *JPEGouldSA3.r* file available as part of these Supplementary Materials, and wait for the user interface to load.

***Maxent settings***

**
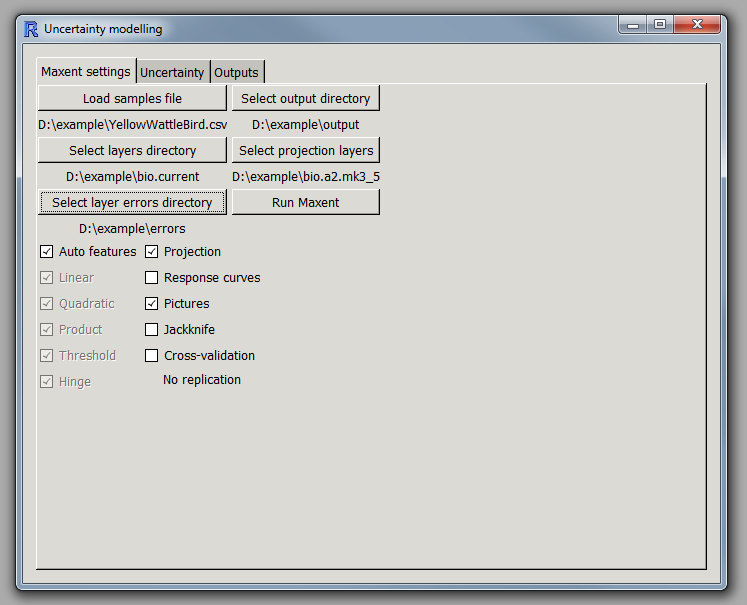
**Using the GUI that has now loaded, select the appropriate input sample file, climate layers directory and output directory by pressing the relevant buttons and choosing a suitable file or folder in the resulting dialog box. Figure 1 below shows what a completed setup might look like.

Figure 1 Example of Maxent settings used to simulate uncertainty in the Yellow Wattlebird using Tasmanian climate information.

**Optionally,** if you wish to project results using future climate information, check the “Projection” box and then click on the “Select projection layers” button to select a directory containing layers. Note that the filenames must be *identical* to the corresponding climate layers in the original layers directory, or Maxent will return an error.

**Also optionally,** if you wish to model climatic uncertainty (called “Environmental error” in the program), click on the “Select layer errors directory” button to select a directory containing layers. These must be of the form sXXmYY.asc and contain minimum (XX = 01) and maximum (XX = 02) temperature data as well as rainfall (XX = 03) for each month (YY = 01 to 12). The resolution does *not* need to be the same as the current and projected climate layers, and in fact should be much coarser (see the main article for details).

### Single Maxent run

If you wish to run Maxent without uncertainty, click the “Run Maxent” button – this will call Maxent with the settings described. Once it is finished you can open a html-formatted Maxent report located in the output folder, as would be the case running Maxent by itself. Note that you cannot access single Maxent runs from the Outputs pane of the main program.

### Uncertainty modelling

Select the sources of uncertainty to model and for each source define the sample size and parameter values, then press “Run Maxent with uncertainty” to run through the simulation. Figure 2 below demonstrates the settings used in our article – note that if you use these settings with the data provided, it will take a long time to run!

**
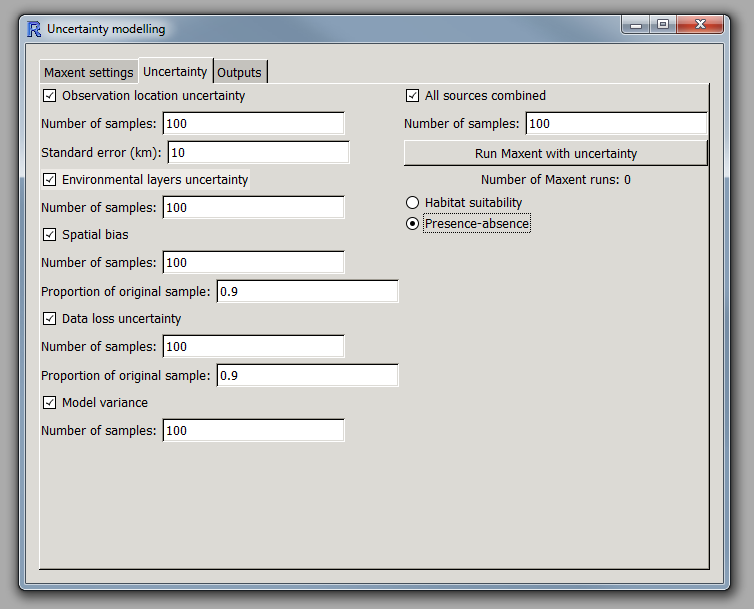
**

Figure 2 Example of settings used to simulate uncertainty in the previously selected species and climate data.

## Examining output

Select the desired error source, species and climate projection from the dropdown menus. Use the “Previous”, “Next”, “Mean” and “Standard deviation” buttons to navigate the results.


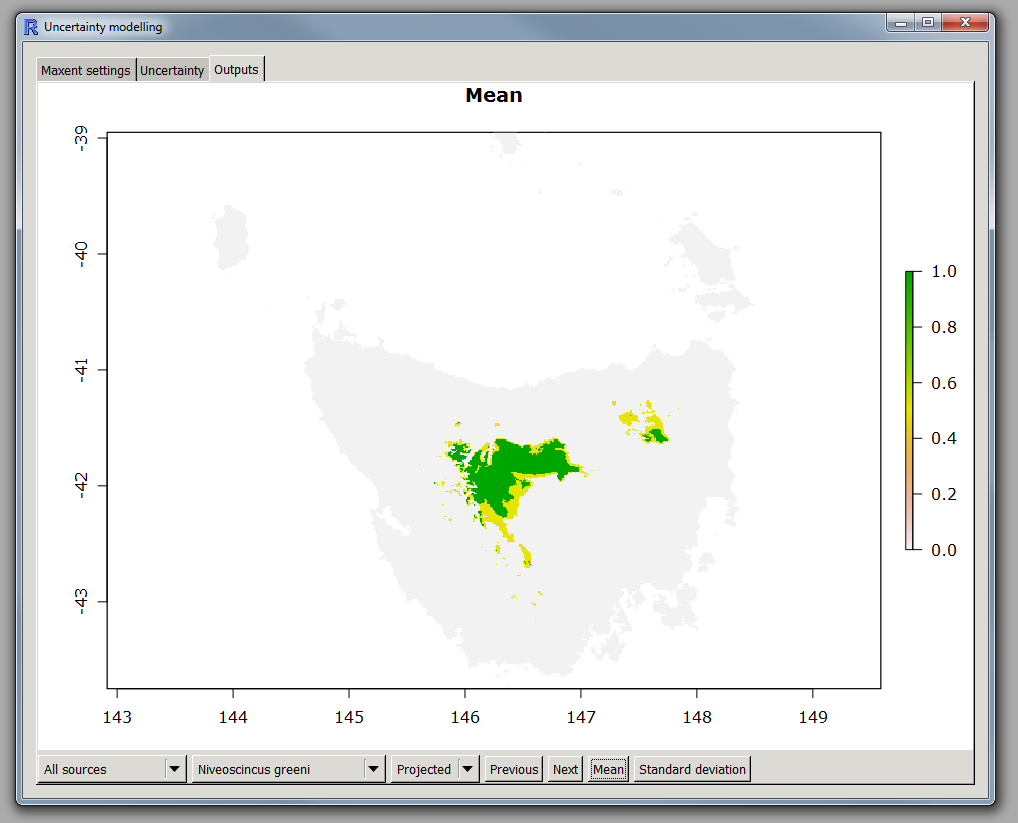


Figure 3 Example of output from an uncertainty simulation, in this case showing the proportion of runs that predicted presence versus absence.
